# Parapoxvirus Infections of Red Deer, Italy

**DOI:** 10.3201/eid1704.101454

**Published:** 2011-04

**Authors:** Alessandra Scagliarini, Francesca Vaccari, Filippo Turrini, Alessandro Bianchi, Paolo Cordioli, Antonio Lavazza

**Affiliations:** Author affiliations: Alma Mater Studiorum Università di Bologna, Bologna, Italy (A. Scagliarini, F. Vaccari, F. Turrini);; and Istituto Zooprofilattico Sperimentale della Lombardia e dell’Emilia Romagna, Brescia, Italy (A. Bianchi, P. Cordioli, A. Lavazza)

**Keywords:** Red deer, Chordopoxvirinae, parapoxvirus, evolution, zoonoses, viruses, Italy, dispatch

## Abstract

To characterize parapoxviruses causing severe disease in wild ruminants in Stelvio Park, Italy, we sequenced and compared the DNA of several isolates. Results demonstrated that the red deer isolates are closely related to the parapox of red deer in New Zealand virus.

The genus *Parapoxvirus* (family *Poxviridae*, subfamily *Chordopoxvirinae*) comprises several members: orf virus (OV), bovine papular stomatitis virus (BPSV), pseudocowpox virus (PCPV), and parapox of red deer in New Zealand virus (PVNZ). PVNZ is responsible for a contagious pustular dermatitis in farmed red deer, with outbreaks reported only in New Zealand (*1*). Cases of parapoxvirus (PPV) pustular stomatitis were reported in wild ruminants in Stelvio Park in the Italian Alps during 2008. The affected animals had erosions and ulcers in the mouth, which led to death by starvation, particularly during the winter. Similar cases have also been described during 1992 in Finland and Norway in reindeer (*Rangifer tarandus*). Recently, the causative viruses of the clinical forms in reindeer were shown to be closely related to OV virus and PCPV, excluding the circulation of PVNZ in these countries (*2**,**3*).

To characterize the PPV agents causing severe disease in wild ruminants of Stelvio Park, we sequenced and compared the DNA of several isolates. Results showed that the viruses isolated from chamois (*Rupicapra rupicapra*) and ibex (*Capra ibex*) were closely related to OV, whereas the isolates from red deer (*Cervus elaphus*) grouped with PVNZ. Our findings provide new information about the diffusion of PPVs in wild ruminants and evidence that PVNZ is circulating outside New Zealand.

## The Study

**Figure 1:**
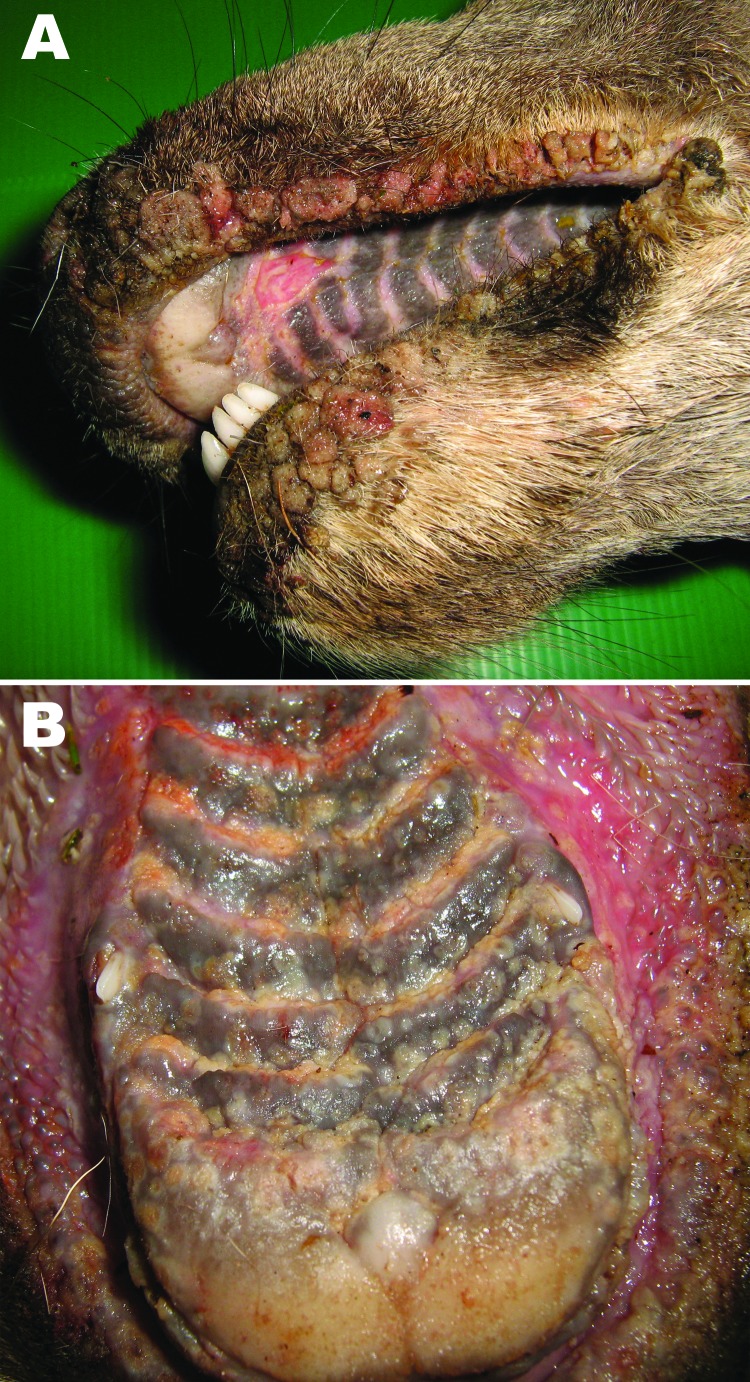
Papular stomatitis in a red deer. A) Proliferative lesions on the lips; B) erosions, vesicles, and ulcers in the mouth.

Cases of a severe contagious stomatitis were reported during winter 2008–09 in wild ruminants of Stelvio Park, Italy. Some affected animals were found dead with proliferative lesions, erosions, and ulcers on the lips and on the hard palate (Figure 1).

Samples collected for pathologic examination from 3 red deer, 2 chamois, and 1 ibex were submitted to Istituto Zooprofilattico della Lombardia e dell’Emilia Romagna (Brescia, Italy) for identification of the causative agent of the disease. Viral particles, identified by morphologic features as PPV, were observed by negative stain electron microscopy in the material collected for pathologic examination from all the affected animals.

After injection into primary lamb keratinocytes (*4*), the viruses showed signs of cytopathic effect after at least 1 week. PCR was used to amplify the PPV *B2L* gene encoding a major envelop protein (*3**,**5*), and a specific amplification product has been obtained from all the isolates. To further characterize the viral DNA, we sequenced the PCR products and compared them with several *Chordopoxvirinae* homologue sequences of field and reference strains (Table). The PPV isolates from chamois and ibex were closely related to OV with 98.2%–99.3% identity at the nt level and 97.3%–98.4% identity at the aa level, compared with the reference strain OV NZ2. These results confirm little or no variations between PPVs that originate from different animal species and from different geographic areas as we and others already have reported (*2**–**6*).

**Table:** Chordopoxviruses used for the phylogenetic analyses of the major envelope protein sequences*

Chordopoxvirus species	Original host	GenBank accession no.
PVNZ 168/09	Red deer	HQ239068
PVNZ 348/08	Red deer	HQ239070
PVNZ 256/08	Red deer	HQ239069
OV 257/09	Chamois	HQ239071
OV 373/08	Ibex	HQ239072
OV 485/09	Chamois	HQ239073
PCPV BO35	Bovine	AY453653
PCPV F00.128R	Reindeer	AY453657
PCPV F00120.R	Reindeer	GQ329669
PCPV F00.91.R	Reindeer	AY453658
PCPV VR634	Human	GQ329670
PCPV F99.177C	Bovine	AY453663
OV NZ-2	Sheep	U06671
OV IA82	Sheep	AY386263
OV F92.849	Reindeer	AY453659
OV Orf11	Sheep	AY453666
OV AICHI	Japanese serow	AB521165
OV SA00	Goat	AY386264
OV D1701	Sheep	AY453654
PVNZ RD86	Red deer	AY453655
BPSV Aomori	Bovine	AB044797
BPSV Chiba	Bovine	AB044798
BPSV V660	Bovine	AY453664
SPV	Seal	AF414182
CPXV ref strain BR	ATCC VR302	AF482758
MPXV	Human	AF380138
HPXV	Horse	DQ792504
VACV ref strain WR	ATCC VR1354	NC 006998
CMLV	Camel	AF438165
VARV	Human	L22579
SPPV	Sheeppox virus	AF199594
LSDV	Bovine	NC_003027
DPV	Mule deer	NC_006967
YLDV	Monkey	NC_002642
MOCV	Human	U60315
FWPV	Bird	AF198100

*PVNZ, parapox of red deer in New Zealand virus; OV, orf virus; PCPV, pseudocowpox virus; BPSV, bovine papular stomatitis virus; SPV, seal poxvirus; CPXV, cowpox virus; ATCC, American Type Culture Collection; MPXV, monkey poxvirus; HPXV, horsepox virus; VACV, vaccinia virus; CMLV, camelpox virus; VARV, variola virus; SPPV, sheeppox virus; LSDV, lumpy skin disease virus; DPV, deerpox virus; YLDV, Yaba like disease virus; MOCV, molluscum contagiosum virus; FWPV, fowlpox virus.

Surprisingly, the *B2L* sequences of the 3 red deer isolates showed 100% identity with that of PVNZ RD86, suggesting that PVNZ could be the cause of the disease. To support this preliminary evidence, we further characterized the red deer viral strains. We amplified the vascular endothelial growth factor (*VEGF*) gene of the red deer isolates (*7*), which enabled us to obtain a specific amplification product from all 3 viral strains. The *VEGF* sequence of strain 348/09 showed 100% identity with PVNZ RD86, and strains 256/08 and 168/09 were 97.8% and 99.6% identical at the nt level and 96.55 and 98.8% identical at the aa level, respectively, which showed that multiple strains are circulating in Stelvio Park.

**Figure 2:**
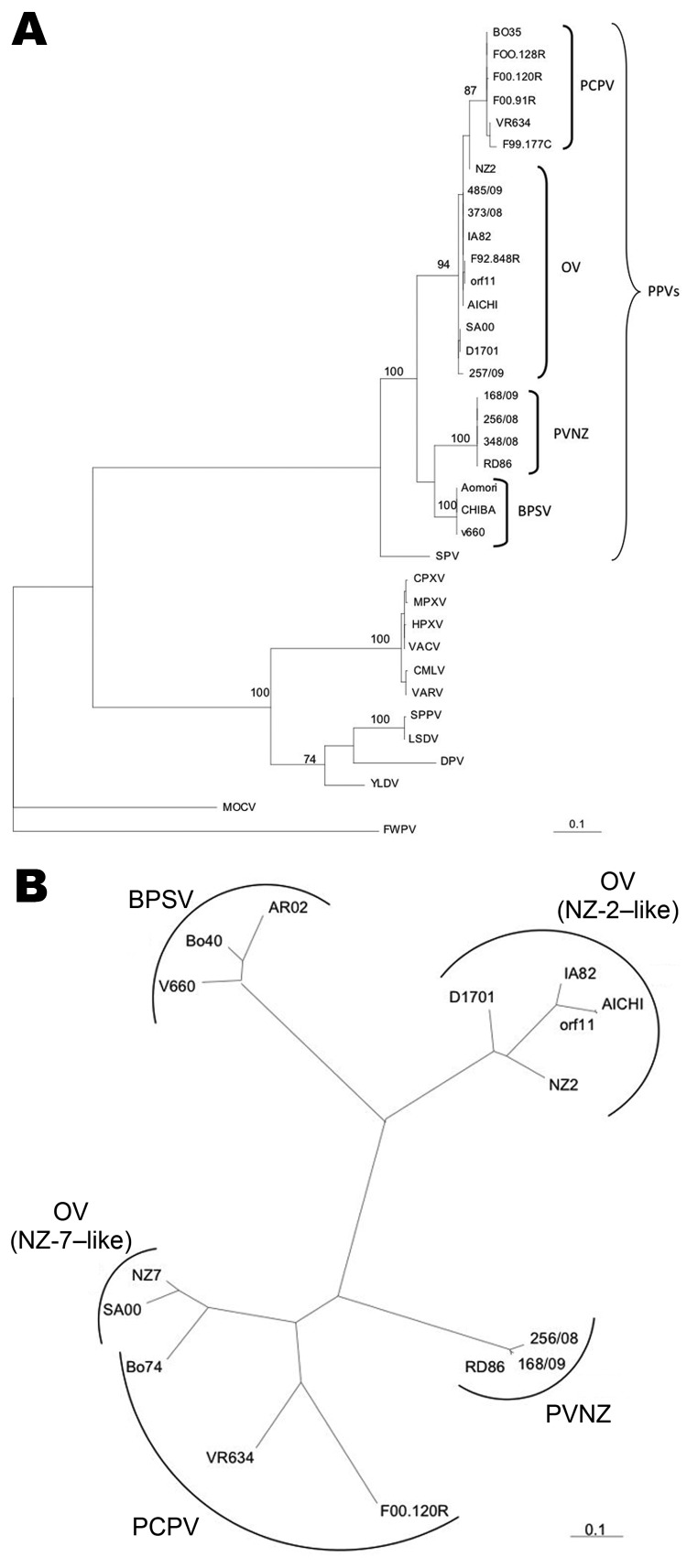
A) Phylogenetic tree of chordopox virus (Table) calculated from the deduced amino acid sequences of the major envelop protein gene. Chordopox virus sequences were edited to correspond to the amino acid sequences of parapoxviruses and aligned by using ClustalW (www.ebi.ac.uk/clustalw). Analyses were performed by using PHYLIP version 3.69 (distributed by J. Felsenstein, University of Washington, Seattle, WA, USA) and the maximum-likelihood method. Numbers on the nodes show the percentage of bootstrap calculated for 1,000 replicates. B) Phylogenetic tree based on the amino acid sequences of the parapoxviral vascular endothelial growth factor (*VEGF*) gene sequences. The genetic distance was estimated by using the Jones-Taylor-Thornton model of the program PRODIST and the phylogenies by using the Fitch-Margoliash method of FITCH (www.phylip.com). Tree was constructed by using PHYLIP version 3.69. Strain 348/08 is not shown because it shares 100% identity with RD-86. Scale bars indicate amino acid substitutions per site.

The classification of PVNZ as a new species of PPV originally was based on comparisons of restriction endonuclease digestion profiles and DNA hybridization analysis (*8*). Sequence analysis performed on the 2 genes of the 3 red deer isolates of Stelvio Park, confirmed the already reported genetic distance between PVNZ and the other PPV species (*3**,**7*). In particular, the phylogenetic analysis performed on the *B2L* gene showed that PVNZ sequences are closely related to BPSV (Figure 2, panel A), whereas the analyses conducted on the *VEGF* gene demonstrated a greater similarity between PVNZ, PCPV, and NZ-7–like VEGF variants (Figure 2, panel B). The latter analysis contributes to clarify the sequence relatedness between the 3 PPV species at the level of this gene, which further supports the hypothesis that PVNZ *VEGF* could have been acquired by natural recombination (*7*).

## Conclusions

We demonstrated that the outbreaks of papular stomatitis in wild ruminants from Stelvio Park resulted from different PPV species: OV is the causative agent in chamois and ibex, and PVNZ caused the disease in red deer. Our results confirm that wild ruminants are susceptible to OV (*4*). This additional observation seems to prove that viruses isolated from chamois and ibex represent an adaptation of the same virus infecting domestic species rather than a separate viral species, despite the fact that the cause of contagious ecthyma in chamois is still considered a tentative species among the PPV genus by official taxonomy (*9*). In the cases reported here, no obvious connections existed between the disease in domestic and wild population, but transmission of PPV from sheep and goats to chamois and ibex cannot be excluded. PPVs are highly contagious and able to be transmitted either by direct contact between animals or indirectly by environmental contamination (*10*).

The genetic characterization of the strains isolated from red deer confirms the presence of PVNZ in Italy. This virus was first noted in New Zealand in 1986 (*1*). Red deer were brought into New Zealand from Europe around 1850, and PVNZ has been speculated to have been introduced from the old continent (*8*). Until now, the disease has never been reported outside New Zealand.

The clinical signs described in New Zealand red deer are generally mild, with lesions limited to the skin and to the epithelial surface of the growing antlers (velvet). In the cases reported here, the disease was severe enough to cause the deaths of the animals. We cannot exclude that mild forms also can occur in wild red deer, and that only the most severe cases of the disease might have been brought to our attention; for this reason, additional data are needed to elucidate the clinical features of the disease in Italy. No information is available about the possibility of PVNZ natural transmission to domestic species, but inoculation of the virus into OV-naive sheep produced milder lesions than did OV (*8*). Given the genetic similarity between the PPVs that are infecting red deer and cattle, ecologic studies should be designed to evaluate the susceptibility of these animal species, respectively, to PCPV, BPSV, and PVNZ.

Most PPVs are transmissible to humans, and these infections share clinical manifestations and exposure risks with other, potentially life-threatening zoonoses (*11*). The transmission of PPVs from deer to humans already has been reported (*12**,**13*); for these reasons, we cannot rule out that PVNZ could be transmitted to humans as a consequence of wildlife activities and manipulation of carcasses.
